# 5-Aminosalicylic Acid Prevents Disease Behavior Progression and Intestinal Resection in Colonic and Ileocolonic Crohn's Disease Patients: A Retrospective Study

**DOI:** 10.1155/2021/1412663

**Published:** 2021-08-09

**Authors:** Jian Wan, Xuan Wang, Yujie Zhang, Xianmin Xue, Yani Li, Zhenzhen Liu, Shuang Han, Min Chen, Yan Nie, Yongquan Shi, Jie Liang, Kaichun Wu

**Affiliations:** ^1^State Key Laboratory of Cancer Biology, National Clinical Research Center for Digestive Diseases and Xijing Hospital of Digestive Diseases, Fourth Military Medical University, Xi'an, Shaanxi Province, China; ^2^Department of Neurology, Xijing Hospital, Fourth Military Medical University, Xi'an, Shaanxi Province, China; ^3^Department of Histology and Embryology, School of Basic Medicine, Xi'an Medical University, Xi'an, China

## Abstract

**Background and Aims:**

The efficacy of 5-aminosalicylic acid (5-ASA) in the long-term outcome of Crohn's disease (CD) patients was uncertain. This study aimed to evaluate the efficacy of the 5-ASA in preventing disease behavior progression and intestinal resection in CD patients.

**Methods:**

CD patients were prospectively enrolled from January 2008 to September 2019 in Xijing Hospital. Disease behavior progression was defined as the development of stricturing (B2) or penetrating disease (B3) in patients with nonstricturing/nonpenetrating disease (B1) at diagnosis. Cox regression analyses were used to investigate the associations between disease location progression, disease behavior progression, and intestinal resection and multiple covariates.

**Results:**

In total, 122 CD patients were followed up for 4.3 years. At the time of diagnosis, disease location was ileal in 19.7% (24/122), colonic in 41.0% (50/122), and ileocolonic in 39.3% (48/122). A total of 87 (71.3%) patients had B1 at diagnosis. The disease behavior progression and intestinal resection rates were 42.5% (37/87) and 29.5% (36/122). The use of 5-ASA reduced the risk of disease behavior progression (HR 0.30, 95% CI 0.14–0.61, *P* = 0.001) and intestinal resection (HR 0.33, 95% CI 0.17–0.90, *P* = 0.027) in colonic and ileocolonic CD patients. Patients who presented with ileal disease at diagnosis did not have the same protective effects when taking 5-ASA (*P* > 0.05).

**Conclusions:**

The use of 5-ASA could improve the long-term outcome of CD patients with colon involvement. The result emphasized the importance of early use of 5-ASA in the daily management of colonic involved CD.

## 1. Introduction

Crohn's disease (CD) is an inflammatory bowel disease (IBD) that may involve the whole gastrointestinal tract [1]. The incidence of CD has increased sharply in China [1, 2]. CD is a progressive and dynamic disease that leads to bowel damage and disability [3].

Population-based studies demonstrate that disease location is relatively stable in CD patients and is presented with ileal, ileocolonic, or colonic disease in about one-third each [3]. Only about 6.5%–13.5% of the patients experience a change in disease location [4]. A multicenter prospective disease registry study in China demonstrates that more than half of the CD patients were presented with ileocolonic disease at diagnosis and the rates of ileal and colonic disease were 27.8% and 14.4% [5]. Furthermore, about 56%–81% of CD patients have inflammatory disease behavior at diagnosis, whereas about 5%–25% present with stricturing or penetrating disease behavior [3, 4]. Cumulative risk of developing stricturing or penetrating disease among those patients with inflammatory behavior is 18% at 7 years [6] and 51% at 20 years [7] after diagnosis. Risk factors associated with developing stricturing or penetrating disease include young age at diagnosis [8], change in disease location [6], ileal/ileocolonic involvement, and penetrating or stricturing disease phenotype [9]. However, these factors have poor precision as predictors and are not widely accepted as accurate predictors [1]. There is a high rate of surgery in the CD patients. The cumulative risk of surgery 10 years after diagnosis was about 40–55% based on studies conducted in Western countries [3, 4].

Current strategies of CD treatment usually focus on the induction and maintenance of remission, prevention of complications and disease progression, and reducing the risk of surgery [1]. In the past, the initial treatment for CD patients was always the use of 5-aminosalicylic acid (5-ASA). Although the recent European Crohn's and Colitis Organization (ECCO) consensus has suggested against the use of 5-ASA for induction and maintenance of remission of Crohn's disease [10], 5-ASA is still widely used for the treatment of CD, especially in patients who presented with colonic or ileocolonic disease location, inflammatory disease behavior, and mild disease activity [11–13]. In a Swiss IBD Cohort, 59% of the CD patients were treated with 5-ASA before [13]. Forty-seven percent of children and adolescent CD patients were treated with 5-ASA during their disease course [14]. In China, about 58%–73% of the CD patients have been treated with 5-ASA before, especially 35% using 5-ASA as the only medicine [15–17]. As the most widely used medicine for CD [18], efficacy and suitable subtype of CD for the use of 5-ASA would be of importance for investigation. Many studies focused on the efficacy of 5-ASA in CD patients for the induction remission, prevention of relapse, and maintenance of surgically induced remission [19] but the results were conflicting and uncertain [20]. Some studies showed that sulfasalazine might have efficacy in remission induction in colonic CD [21, 22]. Since the isolated colonic CD was thought to be quite different from CD with small intestinal involvement [23], it can be speculated that 5-ASA might just only have efficacy in colonic involved CD patients, but not in all the CD patients. In addition, few studies focused on the efficacy of 5-ASA in the long-term outcome of CD patients, such as the disease location and behavior progression and intestinal resection.

In this study, we aimed to evaluate the use of the 5-ASA in reducing the risk of disease progression and surgery in CD patients, especially in the colon involved patients.

## 2. Methods

### 2.1. Study Population and the Endpoint of Study

The CD patients who were diagnosed in the Department of Digestive Disease, Xijing Hospital, and followed up at the specialist clinic for IBD from January 2008 to September 2019 were prospectively recruited into a database. The staffs of the IBD clinic include experienced gastroenterologists, gastrointestinal specialist pathologists, radiologists, nurses, specialist surgeons, and nutritionists. CD was diagnosed based on the medical history and clinical manifestations, combined with the endoscopic and histological findings according to the Chinese consensus on diagnosis and treatment in IBD, which agrees with the ECCO consensus [24, 25]. For all patients included in our study, we required a confirmed diagnosis of CD and a follow-up of at least 6 months after the diagnosis. Patients with uncertain diagnosis or incomplete data were excluded.

The primary outcome of our study was the change of behavior. The secondary outcomes were the change of location and intestinal resection. The endpoint of each outcome was defined as the data of change in location, behavior, and intestinal resection during surveillance. If each outcome of the patient did not develop, the patients were censored at the date of the latest available colonoscopy up to September 2019.

### 2.2. Classifications and Definitions

In our study, all the CD patients were categorized according to the Montreal classification [26] both at diagnosis and throughout the follow-up period. Age at diagnosis (A) was categorized as three levels: A1 (<17 years), A2 (17–40 years), and A3 (>40 years). Disease location (*L*) included ileal (*L*1), colonic (*L*2), ileocolonic (*L*3), and upper gastrointestinal diseases (*L*4). Change in disease behavior (*B*) in our study was defined as the development of *B*2 (stricturing) or *B*3 (penetrating) in patients with *B*1 (nonstricturing, nonpenetrating) at diagnosis.

### 2.3. Treatment and Follow-Up Policy

All the CD patients were followed up regularly at the specialist clinic of IBD every 1 to 6 months based on the patients' conditions. Medication was defined as first exposition to a certain treatment. Treatment included 5-ASA (oral or topical), corticosteroid (not included topical corticosteroids), immunomodulators, biologics (infliximab or adalimumab), and intestinal resection. Intestinal resection was defined as the resection of a part of the bowel because of uncontrolled intestinal inflammation or a CD-related complication.

### 2.4. Clinical Data Collection

Medical histories of the study patients were reviewed and clinical data were collected, which include age, age at onset, gender, disease duration, appendicectomy history, Crohn's Disease Activity Index (CDAI), erythrocyte sedimentation rate (ESR), smoking, clinical features, disease location and behavior and perianal disease when diagnosed and during follow-up, each endoscopy result, and treatment. Smoking status was recorded at the time of diagnosis. Current smoking was defined as patients who had smoked at least 6 months before diagnosis, while former smoking was defined as stopping smoking for at least 6 months. The endoscopy reports of every study patient and the associated images were reviewed by experienced endoscopist (Y.Z.). Any disagreements with original record were solved by discussion with another experienced endoscopist (K.W.).

### 2.5. Ethical Considerations

The study was approved by the ethical committee of Xijing Hospital affiliated to the Fourth Military Medical University in Xi'an, China. All of the patients or their legal representatives signed the informed consent form at the time of enrolling in the cohort.

### 2.6. Statistical Methods

The data were analyzed using the SPSS 19.0 (IBM, Armonk, NY, USA) computer software for Windows. Quantitative variables were summarized as median and interquartile range (IQR). Categorical variables were expressed as frequency and percentage (%). Two-tailed *t*-test or Mann–Whitney test was used to compare the continuous variables for data and the chi-square and Fisher's exact test were used to compare the frequencies of categorical variables, as appropriate. Cox regression was used to select risk factors associated with the occurrence of each outcome. The factors which were significant (*P* < 0.10) at the univariate analysis were included in the final cox proportional hazards model. We used hazard ratios (HRs) with 95% confidence intervals (CIs) to quantify the association of the factors with each outcome.

## 3. Results

A total of 141 CD patients were reviewed and 19 patients had incomplete data or no follow-up data. As a result, 122 patients were enrolled in our study. The median follow-up time was 4.3 years (IQR 2.5–7.1 years). Patient characteristics are presented in Table 1. A total of 92 patients had received 5-ASA. No significance was found between patients treated with and without 5-ASA.

### 3.1. Change in Disease Behavior

**Figure 1:**
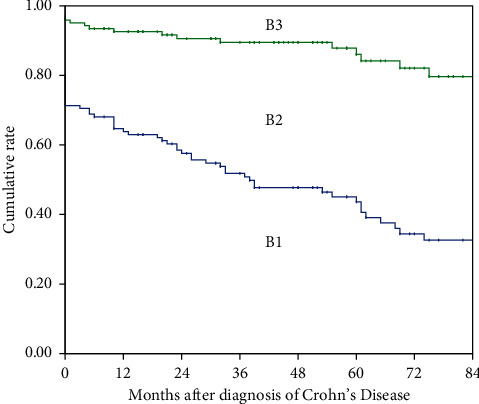
The distribution of disease behavior over the first 7 years of disease. *B*1, nonstricturing, nonpenetrating disease; *B*2, stricturing disease; *B*3, penetrating disease.

**Figure 2:**
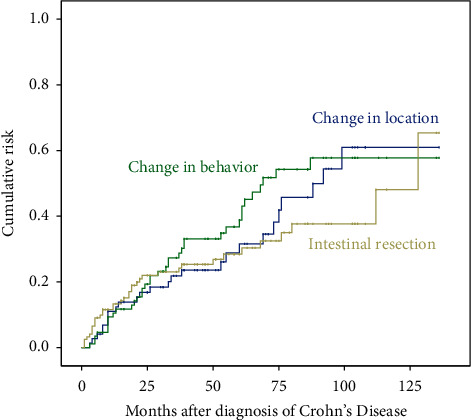
Cumulative risk of change in disease location, disease behavior, and intestinal resection.

Of 87 patients with B1 at diagnosis, 31.0% (27/87) of the patients progressed to *B*2, and 11.5% (10/87) of the patients progressed to *B*3. The distribution of disease behavior over the first 7 years of disease is shown in Figure 1. The median time of change in disease behavior in patients who presented with *B*1 at diagnosis was 30.5 months (IQR 12.8–60.3 months). Of patients with *B*2 at diagnosis, 13.3% (4/30) of the patients progressed to *B*3. The cumulative risk of change in disease behavior in patients who presented with *B*1 at diagnosis is shown in Figure 2. Multivariate analysis showed that factors associated with change in disease behavior in patients with *B*1 at diagnosis were the use of 5-ASA (HR 0.29, 95% CI 0.14–0.58, *P*=0.001) and appendicectomy (HR 0.32, 95% CI 0.12–0.82, *P*=0.018) (Table 2). Biologics treatment was of significance in the univariate analysis (HR 0.43, 95% CI 0.20–0.95, *P* = 0.037) and lost significance in the multivariate analysis (HR 0.47, 95% CI 0.21–1.08, *P* = 0.075) (Table 2).

**Figure 3:**
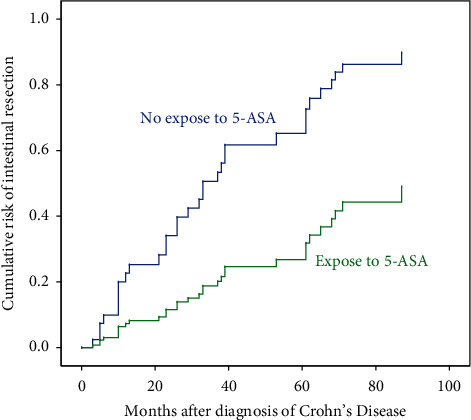
Cumulative risk of change from *B*1 to *B*2/*B*3 in patients with *L*2 and *L*3 at diagnosis. *B*1, nonstricturing, nonpenetrating disease; *B*2, stricturing disease; *B*3, penetrating disease; *L*2, colon location; *L*3, ileocolon location; 5-ASA, 5-aminosalicylic acid.

Subgroup analysis showed that the use of 5-ASA (HR 0.30, 95% CI 0.14–0.61, *P* = 0.001) and appendicectomy (HR 0.30, 95% CI 0.11–0.78, *P* = 0.013) was significantly associated with change to *B*2/*B*3 from *B*1 in patients with *L*2 and *L*3 at diagnosis (Table 2; Figure 3). No factor was significant in the patients presenting with *L*1 at diagnosis (data not shown, *P* > 0.05).

### 3.2. Change in Disease Location

Of the 8 (8/122, 6.6%) patients diagnosed with *L*4, the numbers of patients coexisting with *L*1, *L*2, and *L*3 were 2, 3, and 3, respectively. Changes in disease location were observed in 30 patients (24.6%) during follow-up. Of the patients with *L*1 at diagnosis, 25.0% (6/24) changed to *L*3. Of the patients with *L*2 at diagnosis, 40.0% (20/50) changed to *L*3 and 4.0% (2/50) to *L*2 + *L*4. Of the patients diagnosed with *L*3, only 4.2% (2/48) changed to *L*3 + *L*4. The cumulative risk of change in disease location in *L*1 and *L*2 patients is shown in Figure 2. A total of 26 patients presented with *L*1 and *L*2 at diagnosis changed to *L*3 during follow-up. The median time of change in disease location in these patients was 29.5 months (IQR 10.0–70.0 months). None of the risk factors was significantly associated with change in disease location (Table S1).

### 3.3. Intestinal Resections

A total of 36 (29.5%) patients had intestinal resection in our study. The median time of intestinal resection in all the 122 CD patients was 17.0 months (IQR 5.0–37.8 months). The number of patients who had intestinal resection during the first and second year after diagnosis was 16 (44.4%) and 9 (25.0%). The majority of the 36 patients were *A*2 (47.2%, 17/36) at the time of diagnosis, followed by *A*3 (27.8%, 10/36) and *A*1 (25%, 9/36). Most of the 36 patients presented with *L*2 (41.7%, 15/36) and *L*1 (30.6%, 11/36) at the time of diagnosis, followed by *L*3 (19.4%, 7/36) and *L*4 (8.3%, 3/36). For disease behavior at the time of diagnosis, most patients were *B*1 (63.9%, 23/36), followed by *B*2 (25.0%, 9/36) and *B*3 (11.1%, 4/36). The cumulative risk of intestinal resection is shown in Figure 2. Multivariate analysis showed that risk factors associated with intestinal resection were *B*3 at diagnosis (HR 4.65, 95% CI 1.24–17.34, *P* = 0.022), the use of 5-ASA (HR 0.40, 95% CI 0.20–0.87, *P* = 0.024), and the use of immunomodulators (HR 0.29, 95% CI 0.09–0.88, *P* = 0.030) (Table 3).

Subgroup analysis was performed according to the disease location at diagnosis. The 5-ASA (HR 0.33, 95% CI 0.17–0.90, *P* = 0.027) and biologics (HR 0.24, 95% CI 0.07–0.82,*P* = 0.023) were used as protective factors for intestinal resection in patients who presented with *L*2 and *L*3 at diagnosis. No factor was significant in the patients presenting with *L*1 at diagnosis (Table S2).

## 4. Discussion

Our study demonstrated that the use of 5-ASA could reduce the risk of disease behavior progression and intestinal resection in patients with *L*2 and *L*3 at diagnosis. A cohort of 122 CD patients was followed up with a median of 4.3 years. About a quarter of the patients had a change in disease location. At diagnosis, more than seventy percent of the patients presented with *B*1 and 42.5% of the *B*1 patients progressed to *B*2 or *B*3. About thirty percent of the patients had intestinal resection and most of the resection occurred in the first two years. Patients who presented with *B*3 at diagnosis were associated with higher rate of intestinal resection, while the use of immunomodulators was a protective factor for intestinal resection. Biologics use decreased the risk of intestinal resection in patients with *L*2 and *L*3 at diagnosis.

Disease location at diagnosis was most presented at colitis and ileocolitis, which was similar with a Europe-wide population-based study [27]. However, in an Asia-Pacific region population-based study, about half of the CD patients presented with ileocolitis at diagnosis, and the other two types of location accounted for a quarter each [28]. Previous study had shown that the disease location remained stable [1, 3, 29]. Only 6.5–13.5% of the CD patients had experienced a change in disease location over time [4, 30]. Compared with these studies, our study had a higher rate of change in disease location. Disease behavior of CD changed over time [1, 4]. Results on progression rates varied widely around the world. In a population-based study from Olmsted County, the results showed that the cumulative risk of behavior progression was 33.7% at 5 years and 50.8% at 20 years after diagnosis [7]. The cumulative risk of CD behavior that changed from B1 to B2/B3 was 20.4% in Asia and 16.9% in Australia in 18 months of follow-up in a population-based study [28]. The progression rate of disease behavior was 42.5% with a 4.3 years' follow-up in our study, which was higher than the previous population-based studies. The disease location and behavior progression rates in our study were higher than those in the previous studies. The reason might be that our study was a tertiary hospital-based study while the previous studies were population-based studies, which led to an overrepresentation of severe cases. The patients included in our study might have had more serious disease situation. The progression rate might not represent the real rate of disease behavior progression in China.

The use of 5-ASA in CD is not well established. Previous studies mainly focused on the efficacy of 5-ASA in the remission induction and maintenance of CD patients [19]. It is contradictory whether oral 5-ASA was effective compared with placebo for induction and maintenance of remission in CD patients [3]. Although the ECCO consensus recommended against the use of 5-ASA in CD [10], several meta-analyses showed that only sulfasalazine had modest effect on induction of remission [31, 32] and 5-ASA might reduce the probability of clinical relapse in surgically induced remission CD patients [33, 34]. In the Swiss IBD cohort study, surveyed physicians judged response to 5-ASA treatment as clinically successful in 46% of treatment episodes [13]. Mesalamine was effective at 4 g/day as a monotherapy in treatment of active CD [35] and pentasa reduced the CDAI score in comparison with placebo [36]. As colonic CD patients had different genetics microbiota and serology compared with CD patients with small intestinal involvement [23], the treatment might be different. Only a few studies reported data separately for colonic CD patients. Sulfasalazine was effective in remission induction in colonic CD patients in two trials [21, 22], but only small samples of colonic CD patients were included in these two studies. Our study included a large number of *L*2 and *L*3 patients and focused on the effectiveness in the long-term outcomes, such as disease behavior progression and surgery, instead of remission induction and maintenance. Interestingly, the use of 5-ASA was a protective factor associated with disease behavior progression and intestinal resection in patients presented with *L*2 and *L*3 at diagnosis. But patients who presented with *L*1 at diagnosis did not have the same protective effects when taking 5-ASA. 5-ASA was still widely used in mild CD patients in China [37]. These results emphasized the importance of use 5-ASA in the daily management of colonic involved CD. Early use of 5-ASA may be useful to reduce the rate of disease behavior progression and the surgery in colon involved CD patients. The use of 5-ASA might have some good effects on the long-term outcome of colon involved patients. Large sample of randomized controlled trial was needed to confirm our study result in the colon involved patients.

There were some strengths of the present study. First, our study focused on the efficacy of 5-ASA in the long-term outcome of CD patients. Second, as the efficacy of treatment might be different in CD patients with different disease location, subgroup analysis was performed based on the disease location.

There were also several limitations in our study. First, patients were from a single-center hospital in China. Second, due to the retrospective nature of our study, some potential factors, such as c-reactive protein, endoscopic severity, and histological inflammation score, were not included in our study. In the future, large-sample population-based prospective long-term follow-up studies are needed to confirm our conclusions.

## 5. Conclusions

In conclusion, the use of 5-ASA could reduce the risk of disease behavior progression and intestinal resection in colonic and ileocolonic CD patients. The result emphasized the importance of 5-ASA use in preventing disease behavior progression and intestinal resection in CD patients with colon involvement.

## Figures and Tables

**Table 1:** Characteristics of Crohn's disease patients.

	Results (*n* = 122)	Non-5-ASA (*n* = 30)	5-ASA (*n* = 92)	*P* value
Age, median (IQR)	33.0 (26.0–46.0)	32.5 (20.5–40.3)	33.5 (28.0–46.0)	0.082
Age at diagnosis, median (IQR)	27.0 (21.0–40.0)	28.0 (16.0–39.0)	27.0 (22.0–41.8)	0.184
Female, *n* (%)	50 (41.0)	12 (40.0)	38 (41.3)	0.900
Smoking, *n* (%)				0.330
Never	95 (77.9)	25 (83.3)	70 (76.1)	
Former	2 (1.6)	1 (3.3)	1 (1.1)	
Current	25 (20.5)	4 (13.3)	21 (22.8)	
Age at diagnosis, *n* (%)				0.070
*A*1	17 (13.9)	8 (26.7)	9 (9.8)	
*A*2	76 (62.3)	17 (56.7)	59 (64.1)	
*A*3	29 (23.8)	5 (16.7)	24 (26.1)	
Location at diagnosis, *n* (%)				0.753
*L*1	24 (19.7)	5 (16.7)	19 (20.7)	
*L*2	50 (41.0)	14 (46.7)	36 (39.1)	
*L*3	48 (39.3)	11 (36.7)	37 (40.2)	
*L*4 involvement	8 (6.6)	3 (10.0)	2 (2.2)	0.095
Behaviour at diagnosis, *n* (%)				0.403
*B*1	87 (71.3)	23 (76.7)	64 (69.6)	
*B*2	30 (24.6)	5 (16.7)	25 (27.2)	
*B*3	5 (4.1)	2 (6.7)	3 (3.3)	
Perianal disease at diagnosis, *n* (%)	5 (4.1)	1 (3.3)	4 (4.3)	1.000
Appendicectomy, *n* (%)	31 (25.4)	8 (26.7)	23 (25.0)	0.856
Elevated ESR at diagnosis, *n* (%)	73 (59.8)	19 (63.3)	54 (58.7)	0.653
CDAI at diagnosis, median (IQR)	171.0 (102.0–259.5)	161.0 (100.0–342.0)	173.0 (102.0–246.0)	0.562
Cumulative exposures for treatment, *n* (%)				
5-Aminosalicylic acid	92 (75.4)	—	—	—
Corticosteroid	68 (55.7)	15 (50.0)	53 (57.6)	0.466
Immunomodulators	64 (52.5)	13 (43.3)	51 (55.4)	0.249
Biologics	70 (57.4)	18 (60.0)	52 (56.5)	0.738

IQR, interquartile range; ESR, erythrocyte sedimentation rate; CDAI: Crohn's disease activity index; 5-ASA, 5-aminosalicylic acid.

**Table 2:** Factors associated with change in disease behavior

	All B1 patients (*n* = 87)				(*L*2 + L3) in B1 patients (*n* = 71)			
	Univariate		Multivariate		Univariate		Multivariate	
	*P*	HR (95%CI)	*P*	HR (95%CI)	*P*	HR (95%CI)	*P*	HR (95%CI)
Female	0.387	0.75 (0.39–1.45)	NS		0.418	0.73 (0.35–1.55)	NS	
Smoking								
Former vs. never	0.974	NA	NS		0.984	NA	NA	
Current vs. never	0.736	1.13 (0.55–2.34)	NS		0.796	0.90 (0.40–2.01)	NS	
Age at diagnosis								
*A*2 vs. *A*1	0.246	2.03 (0.61–6.72)	NS		0.224	2.45 (0.58–10.37)	NS	
*A*3 vs. *A*1	0.199	2.36 (0.64–8.74)	NS		0.284	2.46 (0.47–12.74)	NS	
Location at diagnosis								
*L*2 vs. *L*1	0.360	1.53 (0.61–3.82)	NS		NA		NA	
*L*3 vs. *L*1	0.931	1.04 (0.39–2.80)	NS		NA		NA	
*L*4 involvement	0.866	1.08 (0.42–2.78)	NS		0.894	0.93 (0.33–2.66)	NS	
Perianal disease	0.398	1.68 (0.51–5.57)	NS		0.241	2.07 (0.62–6.96)	NS	
Appendicectomy	**0.007**	**0.27 (0.11–0.70)**	**0.018**	**0.32 (0.12–0.82)**	**0.022**	**0.33 (0.13–0.85)**	**0.013**	**0.30 (0.11–0.78)**
Elevated ESR at diagnosis	0.559	1.22 (0.63–2.33)	NS		0.172	1.65 (0.80–3.38)	NS	
CDAI at diagnosis	0.180	1.00 (1.00–1.01)	NS		0.115	1.00 (1.00–1.01)	NS	
5-Aminosalicylic acid	**≤0.001**	**0.29 (0.15–0.55)**	**0.001**	**0.29 (0.14–0.58)**	**0.002**	**0.32 (0.16–0.66)**	**0.001**	**0.30 (0.14–0.61)**
Corticosteroid	0.140	0.60 (0.31–1.18)	NS		0.633	0.84 (0.42–1.71)	NS	
Immunomodulators	**0.085**	**0.52 (0.25–1.10)**	0.854	0.93 (0.41–2.11)	0.179	0.59 (0.27–1.28)	NS	
Biologics	**0.037**	**0.43 (0.20–0.95)**	0.075	0.47 (0.21–1.08)	0.130	0.54 (0.24–1.20)	NS	

HR, hazard ratio; ESR, erythrocyte sedimentation rate; NA, not available; NS, not significance (*P* > 0.10) in the univariate Cox regression; CDAI: Crohn's disease activity index.

**Table 3:** Factors associated with intestinal resection.

	All patients (*n* = 122)				(*L*2 + *L*3) patients (*n* = 98)			
	Univariate		Multivariate		Univariate		Multivariate	
	*P*	HR (95%CI)	*P*	HR (95%CI)	*P*	HR (95%CI)	*P*	HR (95%CI)
Female	0.105	1.73 (0.89–3.36)	NS		0.170	1.75 (0.79–3.91)	NS	
Smoking								
Former vs. never	0.809	1.28 (0.17–9.44)	NS		NA		NA	
Current vs. never	0.658	0.83 (0.36–1.90)	NS		0.757	1.15 (0.47–2.79)	NS	
Age at diagnosis								
*A*2 vs. *A*1	**0.047**	**0.44 (0.20–0.99)**	0.301	1.70 (0.62–4.63)	0.507	0.69 (0.23–2.09)	NS	
*A*3 vs. *A*1	0.373	0.66 (0.26–1.65)	0.094	2.15 (0.87–6.34)	0.663	1.33 (0.37–4.76)	NS	
Location at diagnosis								
*L*2 vs. *L*1	0.594	0.81 (0.38–1.73)	0.737	0.85 (0.33–2.18)	NA		NA	
*L*3 vs. *L*1	**0.044**	**0.40 (0.16–0.98)**	0.713	0.82 (0.28–2.39)	NA		NA	
*L*4 involvement	0.979	1.02 (0.31–3.33)	NS		0.924	1.07 (0.25–4.58)	NS	
Behavior at diagnosis								
*B*2 vs. *B*1	0.362	1.44 (0.66–3.15)	0.161	1.87 (0.78–4.48)	0.259	1.69 (0.68–4.18)	0.402	1.49 (0.58–3.82)
*B*3 vs. *B*1	**0.002**	**5.59 (1.86–16.71)**	**0.022**	**4.65 (1.24–17.34)**	**0.035**	**5.03 (1.12–22.59)**	0.558	1.58 (0.34–7.32)
Perianal disease	0.410	0.43 (0.06–3.17)	NS		0.597	0.58 (0.08–4.32)	NS	
Appendicectomy	0.654	1.20 (0.55–2.63)	NS		0.154	1.91 (0.78–4.64)	NS	
Elevated ESR at diagnosis	0.896	0.96 (0.49–1.87)	NS		0.538	1.30 (0.57–2.98)	NS	
CDAI at diagnosis	0.420	1.00 (1.00–1.01)	NS		0.142	1.00 (1.00–1.01)	NS	
5-Aminosalicylic acid	**≤0.001**	**0.10 (0.04–0.23)**	**0.024**	**0.40 (0.20–0.87)**	**≤0.001**	**0.05 (0.01–0.17)**	**0.027**	**0.33 (0.17–0.90)**
Corticosteroid	**≤0.001**	**0.13 (0.05–0.33)**	0.130	0.42 (0.14–1.29)	**0.001**	**0.15 (0.05–0.45)**	0.520	0.66 (0.18–2.37)
Immunomodulators	**≤0.001**	**0.11 (0.04–0.28)**	**0.030**	**0.29 (0.09–0.88)**	**≤0.001**	**0.06 (0.01–0.26)**	0.089	0.22 (0.04–1.26)
Biologics	**0.001**	**0.25 (0.11–0.57)**	0.082	0.45 (0.18–1.11)	**0.001**	**0.13 (0.04–0.45)**	**0.023**	**0.24 (0.07–0.82)**

HR, hazard ratio; ESR, erythrocyte sedimentation rate; NA, not available; NS, bot significance (*P* > 0.10) in the univariate Cox regression; CDAI: Crohn's disease activity index.

## Data Availability

The datasets used and/or analyzed during the current study are available from the corresponding author upon reasonable request.

## Supplementary Materials

Supplementary Materials Table S1. Factors associated with change in location in both *L*1 and *L*2 patients (*n* = 74). Table S2. Factors associated with intestinal resection in *L*1 patients (*n* = 24).
