# Supplementary material for: 5-Aminosalicylic Acid Prevents Disease Behavior Progression and Intestinal Resection in Colonic and Ileocolonic Crohn's Disease Patients: A Retrospective Study
Source: Can J Gastroenterol Hepatol. 2021 Aug 9;2021:1412663. doi: 10.1155/2021/1412663 (PMC8371663; doi:10.1155/2021/1412663)
Supplement: Supplementary Materials — Table S1. Factors associated with change in location in both L1 and L2 patients (n = 74). Table S2. Factors associated with intestinal resection in L1 patients (n = 24). [file 1412663.f1.docx]

| Table S1. Factors associated with change in location in both L1 and L2 patients (n=74) | | | | |
| --- | --- | --- | --- | --- |
|  | Univariate | | Multivariate | |
|  | P | HR (95%CI) | P | HR (95%CI) |
| Female | 0.929 | 1.04 (0.48-2.26) | NS |  |
| Smoking |  |  |  |  |
| Former vs Never | 0.935 | 1.09 (0.14-8.20) | NS |  |
| Current vs Never | 0.586 | 0.77 (0.31-1.95) | NS |  |
| Age at diagnosis |  |  |  |  |
| A2 vs A1 | 0.941 | 0.96 (0.36-2.57) | NS |  |
| A3 vs A1 | 0.909 | 0.94 (0.31-2.81) | NS |  |
| L4 involve | 0.593 | 0.67 (0.16-2.86) | NS |  |
| Behavior at diagnosis |  |  |  |  |
| B2 vs B1 | 0.348 | 1.56 (0.61-3.98) | NS |  |
| B3 vs B1 | 0.121 | 2.68 (0.77-9.32) | NS |  |
| Perianal disease | 0.108 | 2.79 (0.80-9.74) | NS |  |
| Appendicectomy | **0.063** | **0.32 (0.09-1.00)** | 0.264 | 0.49 (0.14-1.71) |
| Elevated ESR at diagnosis | 0.361 | 1.46 (0.65-3.30) | NS |  |
| CDAI at diagnosis | 0.210 | 1.00 (1.00-1.01) | NS |  |
| 5-aminosalicylic acid | **0.084** | **0.50 (0.22-1.10)** | 0.335 | 0.67 (0.29-1.52) |
| Corticosteroid | **0.004** | **0.23 (0.09-0.63)** | 0.120 | 0.41 (0.13-1.26) |
| Immunomodulators | **0.016** | **0.23 (0.07-0.76)** | 0.361 | 0.53 (0.13-2.09) |
| Biologics | **0.047** | **0.34 (0.12-0.99)** | 0.332 | 0.57 (0.18-1.78) |
| HR, hazard ratio; ESR, erythrocyte sedimentation rate; NA, not available; NS, not significance (P>0.10) in the univariate Cox regression; CDAI: Crohn's Disease Activity Index | | | | |

| Table S2. Factors associated with intestinal resection in L1 patients (n=24). | | | | |
| --- | --- | --- | --- | --- |
|  | Univariate | | Multivariate | |
|  | P | HR (95%CI) | P | HR (95%CI) |
| Female | 0.756 | 1.22 (0.36-4.17) | NS |  |
| Smoking |  |  |  |  |
| Former vs Never | 0.987 | NA | NS |  |
| Current vs Never | 0.712 | 0.68 (0.09-5.32) | NS |  |
| Age at diagnosis |  |  |  |  |
| A2 vs A1 | **0.091** | **0.28 (0.06-1.23)** | 0.255 | 4.02 (0.37-44.07) |
| A3 vs A1 | **0.048** | **0.26 (0.07-0.99)** | 0.866 | 1.17 (0.19-7.25) |
| L4 involve | 0.870 | 0.84 (0.11-6.60) | NS |  |
| Behavior at diagnosis |  |  |  |  |
| B2 vs B1 | 0.851 | 0.86 (0.18-4.17) | 0.809 | 1.34 (0.13-14.20) |
| B3 vs B1 | **0.047** | **5.57 (1.02-30.30)** | 0.087 | 7.78 (0.74-81.39) |
| Perianal disease | 0.646 | 0.05 (0.00-2.32E4) | NS |  |
| Appendicectomy | 0.254 | 0.30 (0.04-2.38) | NS |  |
| Elevated ESR at diagnosis | 0.263 | 0.51 (0.15-1.67) | NS |  |
| CDAI at diagnosis | 0.110 | 0.99 (0.98-1.00) | NS |  |
| 5-aminosalicylic acid | **0.050** | **0.29 (0.09-1.00)** | 0.759 | 0.70 (0.07-6.64) |
| Corticosteroid | **0.023** | **0.09 (0.01-0.71)** | 0.094 | 0.06 (0.00-1.61) |
| Immunomodulators | **0.082** | **0.31 (0.08-1.16)** | 0.200 | 0.33 (0.06-1.81) |
| Biologics | 0.719 | 0.78 (0.21-2.97) | NS |  |
| HR, hazard ratio; ESR, erythrocyte sedimentation rate; NA, not available; NS, not significance (P>0.10) in the univariate Cox regression; CDAI: Crohn's Disease Activity Index | | | | |
